# Antenatal multiple micronutrient supplements versus iron‐folic acid supplements and birth outcomes: Analysis by gestational age assessment method

**DOI:** 10.1111/mcn.13509

**Published:** 2023-03-31

**Authors:** Filomena Gomes, Sufia Askari, Robert E. Black, Parul Christian, Kathryn G. Dewey, Martin N. Mwangi, Ziaul Rana, Sarah Reed, Anuraj H. Shankar, Emily R. Smith, Alison Tumilowicz

**Affiliations:** ^1^ The New York Academy of Sciences New York City New York USA; ^2^ NOVA Medical School Universidade NOVA de Lisboa Lisboa Portugal; ^3^ Sight and Life Foundation Basel Switzerland; ^4^ Johns Hopkins Bloomberg School of Public Health Baltimore Maryland USA; ^5^ Department of Nutrition University of California, Davis Davis California USA; ^6^ The Micronutrient Forum Washington District of Columbia USA; ^7^ The Bill & Melinda Gates Foundation Seattle Washington USA; ^8^ Nuffield Department of Medicine University of Oxford Oxford UK; ^9^ Summit Institute for Development Mataram Indonesia; ^10^ Milken Institute School of Public Health The George Washington University Washington District of Columbia USA

**Keywords:** birth outcomes, gestational age assessment, iron and folic acid supplements, multiple micronutrient supplements, nutrition, pregnancy, ultrasound

## Abstract

Meta‐analyses consistently have found that antenatal multiple micronutrient supplementation (MMS) compared with iron and folic acid (IFA) alone reduce adverse birth outcomes. In 2020, the World Health Organization (WHO) placed a conditional recommendation for MMS and requested additional trials using ultrasounds to establish gestational age, because the evidence on low birthweight (LBW), preterm birth and small for gestational age (SGA) was considered inconsistent. We conducted meta‐analyses to determine if the effects of MMS on LBW, preterm birth and SGA differed by gestational age assessment method. Using data from the 16 trials in the WHO analyses, we calculated the effect estimates of MMS versus IFA on birth outcomes (generic inverse variance method and random effects model) stratified by method of gestational age assessment: ultrasound, prospective collection of the date of last menstrual period (LMP) and confirmation of pregnancy by urine test and recall of LMP. The effects of MMS versus IFA on birthweight, preterm birth and SGA appeared consistent across subgroups with no evidence of subgroup differences (*p* > 0.05). When limited to the seven trials that used ultrasound, the beneficial effects of MMS were demonstrated: risk ratios of 0.87 (95% confidence interval [CI] 0.78–0.97) for LBW, 0.90 (95% CI, 0.79–1.03) for preterm birth and 0.9 (95% CI, 0.83–0.99) for SGA. Sensitivity analyses indicated consistency in the results. These results, together with recent analyses demonstrating comparable effects of MMS (vs. IFA) on maternal anaemia outcomes, strengthen the evidence to support a transition from IFA to MMS programmes in low‐ and middle‐income countries.

## Key messages

- World Health Organization (WHO) has a conditional recommendation for antenatal multiple micronutrient supplementation (MMS) noting ultrasound to assess gestational age should be used in future trials.
- These meta‐analyses of the 16 trials informing the WHO guidelines showed that the benefits of MMS versus iron and folic acid on low birthweight, preterm birth and small‐for‐gestational‐age were not different in subgroups of trials using different gestational age assessment methods.
- In trials that performed ultrasounds for gestational age assessment, the benefits of MMS on birth outcomes were demonstrated.
- These analyses strengthen the evidence for the transition to MMS in antenatal care programmes in low‐ and middle‐income countries.

## Graphical abstract

World Health Organization (WHO) has a conditional recommendation for antenatal multiple micronutrient supplements (MMS) noting ultrasound to assess gestational age should be used in future trials. These meta‐analyses of the 16 trials informing the WHO guidelines showed that the benefits of MMS versus iron and folic acidsupplements on low birthweight, preterm birth and small for gestational age births were not different in subgroups of trials using different gestational age assessment methods. In trials that performed ultrasounds for gestational age assessment, the benefits of MMS on birth outcomes were demonstrated. These analyses strengthen the evidence for the transition to MMS in antenatal care programmes in low‐ and middle‐income countries.

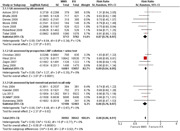

## 1 INTRODUCTION

Micronutrient deficiencies are highly prevalent, affecting two in three women of reproductive age worldwide (Stevens et al., 2022). During pregnancy, the prevalence is likely to be even higher, as requirements for several micronutrients are increased by up to 50% to accommodate maternal and foetal demands (Gernand, Schulze, et al., 2016). The deleterious consequences of micronutrient deficiencies during this critical stage of life and development include low birthweight (LBW), preterm birth, being born small for gestational age (SGA), perinatal mortality, maternal mortality, maternal and child cognitive impairment, premature rupture of membranes, insufficient gestational weight gain and congenital anomalies (Bourassa et al., 2019; Gernand, Schulze, et al., 2016).

Antenatal multiple micronutrient supplements (MMS)—containing 13–15 micronutrients including iron and folic acid (IFA)—are designed to reduce the typically wide gap between the requirements for these essential nutrients and average intakes during pregnancy in low‐ and middle‐income countries (LMICs) (Bourassa et al., 2019; World Health Organization [WHO] et al., 1999). MMS are cost‐effective for reducing adverse birth outcomes in LMICs (Engle‐Stone et al., 2019; Keats et al., 2019, 2022; Smith et al., 2017).

In 2016, the antenatal micronutrient intervention recommended by the WHO for all pregnant women was daily elemental IFA supplements (30–60 mg), while MMS was not recommended (WHO, 2016). Considering the increasing evidence on MMS that emerged subsequently, in 2020 WHO updated the recommendation on using MMS during pregnancy, stating that this intervention was recommended ‘in the context of rigorous research’ (WHO, 2020). This conditional recommendation acknowledged the benefits of MMS but stipulated a need for additional research.

In describing the additional research needed, WHO noted that ‘Evidence on the effects of MMS on the component parts of LBW is inconsistent and controlled clinical trials are needed in which early pregnancy ultrasound is used to establish gestational age with certainty, to understand where the effect on LBW is derived’ (Tuncalp et al., 2020).

Given the desire of the WHO to have results for the effects of MMS from trials that assessed gestational age by ultrasound (WHO, 2020), we conducted additional analyses of the large body of existing data that informed the WHO guidelines. In particular, we aimed to (1) describe methods (ultrasound or other methods) used to determine gestational age in all the trials included for analysis in the 2020 WHO guidelines (WHO, 2020) and (2) determine whether the effect of MMS versus IFA on the three birth outcomes of interest (LBW, preterm birth and SGA) differed according to the method used to assess gestational age in each trial.

## 2 METHODS

When examining the effects of antenatal MMS compared with IFA, the 2020 WHO guidelines (WHO, 2020) relied on data from 16 selected trials (out of 20 trials) of a Cochrane systematic review (Keats et al., 2019). The difference between the 20 trials included in the Cochrane review and the 16 trials included in the 2020 WHO guidelines is due to the slightly different inclusion criteria regarding the intervention and control considered for each analysis. The Cochrane review (Keats et al., 2019) included trials that compared MMS (containing at least three micronutrients) to iron (with or without folic acid) or placebo. Meanwhile, the guideline development group (WHO, 2020) decided to include trials that compared MMS with 13–15 micronutrients to IFA. As two trials compared MMS with placebo, one trial evaluated a supplement with eight micronutrients plus IFA and one trial did not provide folic acid to the control group, only 16 trials contributed data to the updated 2020 WHO guidelines (WHO, 2020). Of these 16 trials, 6 evaluated supplements with 13 or 14 micronutrients and 10 evaluated the well‐established United Nations International Multiple Micronutrient Antenatal Preparation (UNIMMAP) supplements that included 15 micronutrients (WHO et al., 1999). Thus, the WHO analyses were presented in two ways: comparison 1, which compared MMS with 13–15 micronutrients against IFA, and comparison 2, which compared the UNIMMAP supplements against IFA.

For all the 16 trials included in the analyses of the 2020 WHO guidelines, we extracted data on the method used to determine gestational age when women were enrolled in the trial. When this was unclear, we contacted the authors via e‐mail to clarify how gestational age was assessed in their study.

We categorized the methods used for gestational age assessment into:

1. Group 1—gestational age assessed by ultrasound.
2. Group 2—gestational age assessed by prospective collection of the date (1st day) of the last menstrual period (LMP) and confirmation of pregnancy by a urine test.
3. Group 3—gestational age assessed by a recall of the date (1st day) of the LMP.

We then conducted meta‐analyses of the effect of MMS versus IFA on LBW (birthweight less than 2500 g), preterm birth (births before 37 weeks of gestation) and SGA (as defined by the authors of the trials [Keats et al., 2019]), stratified by different methods of gestational age assessment.

For this purpose, we extracted the data on the effect estimates (log risk ratio, standard error) in each study arm for each trial on the three outcomes of interest, and then calculated the pooled effect of MMS versus IFA for each outcome: overall, stratified into three subgroups (groups 1, 2 and 3), and stratified into two subgroups (combining groups 1 + 2, i.e., the two best methods, vs. group 3) of gestational age assessment.

The main analysis included all trials, consistent with 2020 WHO guidelines comparison 1 (MMS vs. IFA, Annex 3, pp. 40–42). Subsequently, we conducted a sensitivity analysis limited to the trials that used UNIMMAP supplements in the intervention arm, following the same trial selection as used for the WHO guidelines (comparison 2, UNIMMAP vs. IFA, pp. 52–53). We repeated the same sensitivity analyses, but added the West trial (West et al., 2014). Comparison 2 of the 2020 WHO guidelines (WHO, 2020) excluded the West 2014 trial because it provided 27 mg of iron and 0.6 mg of folic acid in the MMS arm, while the UNIMMAP supplement contains 30 mg of iron and 0.4 mg of folic acid. However, it should be noted that the actual composition of MMS in the West trial was 28.4–30.2 mg of iron, not meaningfully different from 30 mg, calling into question the removal of a controlled trial with more than 40,000 women.

We used Review Manager (version 5.4) to calculate the overall and subgroup pooled effect estimates (risk ratios [RR] and 95% confidence intervals [CI]) of MMS from individually randomized and cluster‐randomized trials, using the generic inverse variance methods, as per the methodology used for the 2020 WHO guidelines (WHO, 2020). Subgroup differences were assessed with the *χ* ^2^ test for heterogeneity across subgroups, and a *p* value for heterogeneity of <0.05 was considered statistically significant.

## 3 RESULTS

Supporting Information: Appendix Table 1 presents the methods used for gestational age assessment in each of the 16 trials, including the evidence used to support the classification of each study into one of the three groups.

Ultrasound was the most frequent method used to assess gestational age, reported in 7 of the 16 trials (44%) included for analysis (Adu‐Afarwuah et al., 2015; Ashorn et al., 2015; Bhutta et al., 2009; Johnson et al., 2017; Osrin et al., 2005; Persson et al., 2012; Roberfroid et al., 2008). The mean gestational age at enrollment, and the time of the ultrasound scan, varied between 9 and 17 weeks. Four trials (25%) used the date (1st day) of the LMP collected prospectively and confirmation of pregnancy by urine test to determine gestational age (Christian et al., 2003; West et al., 2014; Zagre et al., 2007; Zeng et al., 2008). This method consisted of a pregnancy surveillance system that involved regular visits (e.g., monthly) to the homes of women of reproductive age to ask about LMP dates and conduct urine‐based testing to confirm pregnancies, allowing the identification of pregnancies early in gestation (Gernand, Paul, et al., 2016). Five trials (31%) based gestational age on maternal recall of date (1st day) of LMP (Friis et al., 2004; Kæstel et al., 2005; Liu et al., 2013; Shankar et al., 2008; Sunawang et al., 2009).

A summary of the meta‐analysis results, including overall analyses, subgroup analyses with three groups and subgroup analyses with two groups, is presented in Table 1. The effects of MMS versus IFA on birthweight, preterm birth and SGA were similar across subgroups stratified by the three methods used to assess gestational age, that is, there were no significant differences between subgroups (*p* < 0.05) and the direction of the effect estimates was the same (consistently pointing towards a risk reduction). When limited to the seven trials that used ultrasound for gestational age assessment, MMS versus IFA resulted in a RR of 0.87 (95% CI, 0.78–0.97) for LBW, 0.90 (95% CI, 0.79–1.03) for preterm birth and 0.90 (95% CI, 0.83–0.99) for SGA.

**Table 1:** Summary of results: subgroup analyses of the effect of multiple micronutrient supplementation versus iron and folic acid supplementation on low birthweight, preterm and small for gestational age births, according to method used for gestational age assessment.

Method used for gestational age assessment	Effect of MMS versus IFA on LBW	Effect of MMS versus IFA on preterm birth	Effect of MMS versus IFA on SGA
	RR (95% CI)	RR (95% CI)	RR (95% CI)
	*n* trials	*n* trials	*n* trials
Overall analyses	**0.88 (0.86–0.91)** 16 trials	0.94 (0.88–1.00) 16 trials	0.98 (0.86–1.00) 15 trials
Subgroup analyses (three groups)			
*p* Value (subgroup differences)	0.82	0.23	0.32
1—Ultrasound	**0.87 (0.78–0.97)** 7 trials	0.90 (0.79–1.03) 7 trials	**0.90 (0.83–0.99)** 7 trials
2—Prospective date of LMP collection and confirmation of pregnancy by urine test	**0.89 (0.84–0.95)** 4 trials	0.92 (0.82–1.04) 4 trials	0.98 (0.92–1.05) 4 trials
3—Recall of date of LMP	**0.86 (0.76–0.97)** 5 trials	0.99 (0.99–1.04) 5 trials	0.93 (0.84–1.04) 4 trials
Subgroup analyses (two groups)			
*p* Value (subgroup differences)	0.69	0.06	0.39
1—Best methods (ultrasound and prospective LMP collection)	**0.89 (0.85–0.92)** 11 trials	**0.91 (0.84–0.99)** 11 trials	0.98 (0.96–1.00) 11 trials
2—Recall of date of LMP	**0.86 (0.76–0.97)** 5 trials	0.99 (0.84–1.04) 5 trials	0.93 (0.84–.04) 4 trials

*Note*: Statistically significant results are identified in bold. All *p* values of *χ* ^2^ tests for heterogeneity across subgroups were >0.05.

Abbreviations: IFA, iron and folic acid; LBW, low birthweight; LMP, last menstrual period; MMS, multiple micronutrient supplementation; RR, risk ratio; SGA, small for gestational age.

**Figure 1:**
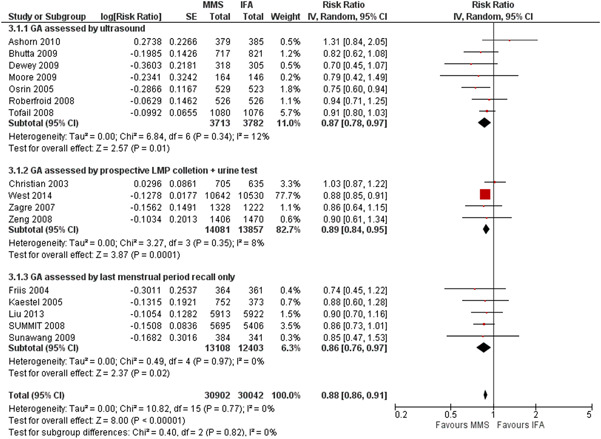
Effect of MMS versus IFA on low birthweight: subgroup analysis by method of gestational age assessment (three groups). CI, confidence interval; GA, gestational assessment; IFA, iron and folic acid supplementation; LMP, (1st day of) last menstrual period; MMS, multiple micronutrient supplementation.

**Figure 2:**
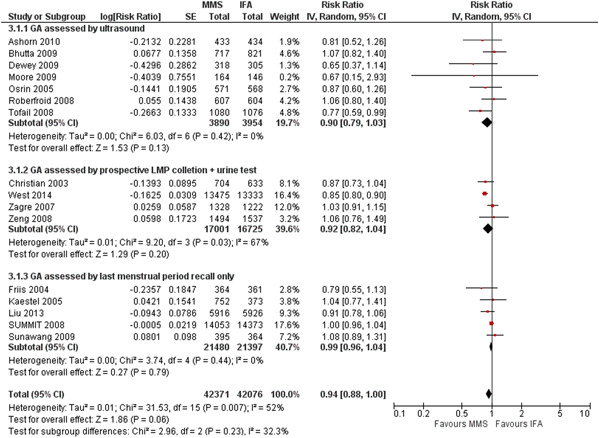
Effect of MMS versus IFA on preterm birth: subgroup analysis by method of gestational age assessment (three groups). CI, confidence interval; GA, gestational assessment; IFA, iron and folic acid supplementation; LMP, (1st day of) last menstrual period; MMS, multiple micronutrient supplementation.

**Figure 3:**
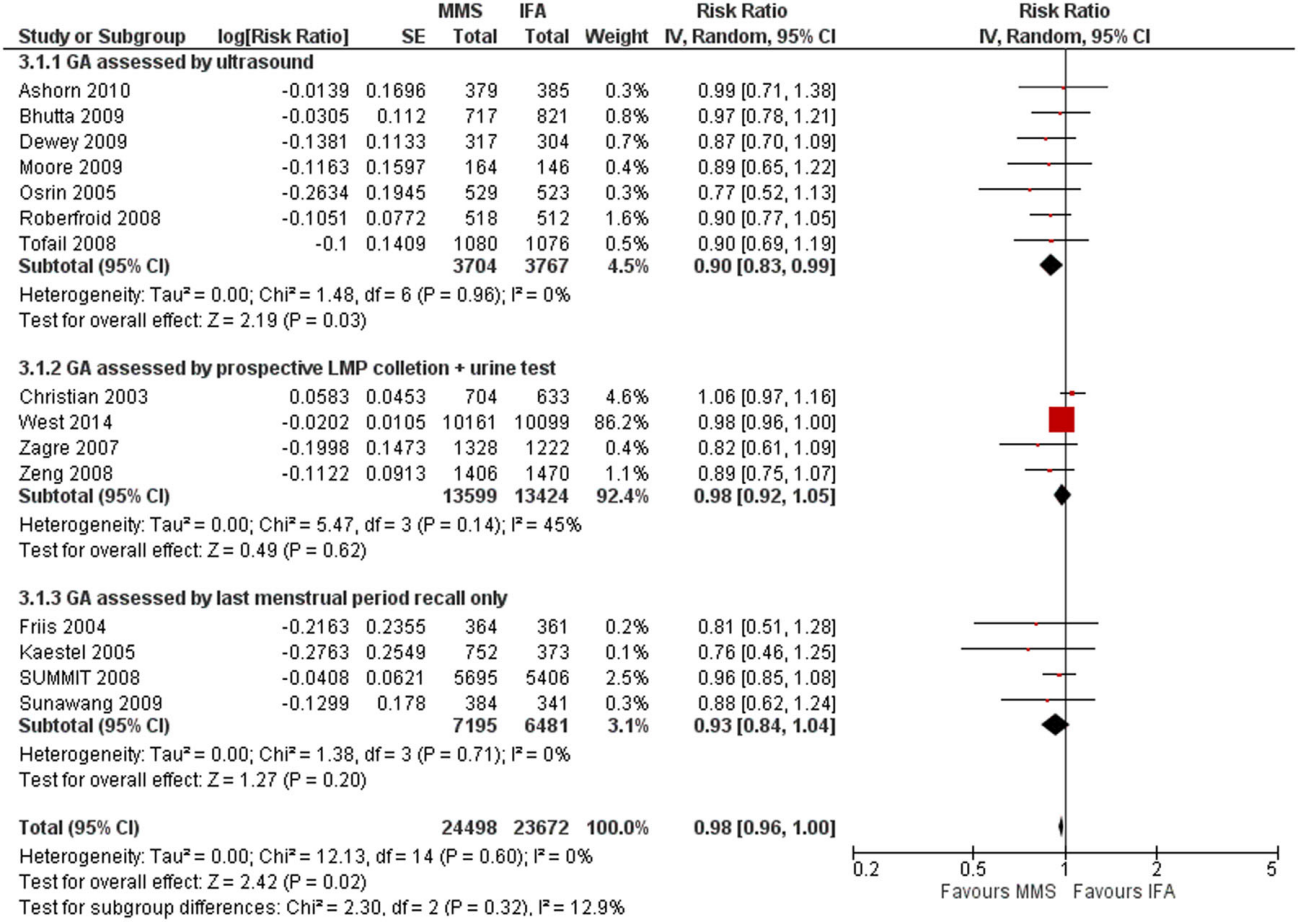
Effect of MMS versus IFA on small for gestational age: subgroup analysis by method of gestational age assessment (three groups). CI, confidence interval; GA, gestational assessment; IFA, iron and folic acid supplementation; LMP, (1st day of) last menstrual period; MMS, multiple micronutrient supplementation.

The two best methods (1 and 2) have a similar RR for preterm birth; when results for these two methods were combined (11 trials), MMS versus IFA resulted in a RR of 0.91 (95% CI, 0.84–0.99) for preterm births, RR of 0.89 (95% CI, 0.85–0.92) for LBW and 0.98 (95% CI, 0.96–1.00) for SGA. The forest plots of the subgroup analyses for each of the three outcomes are presented in Figures 1, 2, 3 (groups 1–3), and Supporting Information: Appendix Figures 1, 2, 3 (groups 1 + 2 vs. group 3).

The results of the sensitivity analyses limited to the trials that provided UNIMMAP supplements in the intervention arm can be found in Supporting Information: Appendix Table 2 (excluding the West et al. trial [West et al., 2014] and Supporting Information: Appendix Table 3 (including the West et al. trial [West et al., 2014]). Results of these sensitivity analyses were consistent with the overall findings, with no significant differences across the subgroups of the gestational age assessment method.

The West 2014 (West et al., 2014) trial observed significant reductions in preterm births (RR, 0.85; 95% CI, 0.80–0.91) and LBW (RR, 0.88; 95% CI, 0.85–0.91), but not SGA births (RR, 0.98; 95% CI, 0.96–1.01). This may explain the trend for a reduction in RR of preterm birth in the sensitivity analyses that include the West trial (Supporting Information: Appendix Table 3), and a null effect with the exclusion of this trial (Supporting Information: Appendix Table 2). It may also explain why the sensitivity analyses without the West trial suggest greater reductions in SGA (RR, 0.91; 96% CI, 0.85–0.98; Supporting Information: Appendix Table 2) than the analyses with the West trial (RR, 0.97; 96% CI, 0.96–0.99; Supporting Information: Appendix Table 3).

The certainty of the evidence contributing to the effect estimates for preterm births and SGA was graded as moderate and for LBW was graded as high (WHO, 2020). The original review (Keats et al., 2019) described the overall risk of bias of the included studies as generally low with at least 50% of the judgements at ‘low risk’ for two domains (allocation concealment and incomplete outcome data) and at least 75% of judgements at ‘low risk’ for the remaining five domains.

## 4 DISCUSSION

The present analyses, based on the data extracted from the 16 trials used for the 2020 WHO guidelines (WHO, 2020), explored the impact of using ultrasound or other methods of gestational age assessment on the effect estimates of MMS versus IFA on the three birth outcomes of interest (LBW, preterm and SGA). We found that:

1. There were three main methods used to assess gestational age, with ultrasound at study enrollment being the most frequently used method.
2. The results of the overall and subgroup analyses for the three outcomes of interest are consistent, pointing towards a risk reduction, across the three methods used to assess gestational age.
3. When the evidence is limited to the trials that used ultrasound—the gold standard for gestational age dating—the beneficial effects of MMS in comparison to IFA are demonstrated (RR of 0.87 [95% CI, 0.78–0.97] for LBW, RR of 0.90 [95% CI, 0.79–1.03] for preterm birth and RR of 0.90 [95% CI, 0.83–0.99] for SGA). This observation also applies to the pooled estimates from the trials that used the two best methods for gestational age assessment (ultrasound and prospective collection of date of LMP with confirmation of pregnancy by urine test), where benefits are evident for preterm birth (RR of 0.91 [95% CI, 0.84–0.99]).
4. When the evidence is limited to the trials that provided UNIMMAP in the intervention arm, the results remained consistent in both sensitivity analyses.

### 4.1 Gestational assessment methods: strengths, limitations and impact on birth outcomes

The most accurate method to establish or confirm gestational age is an ultrasound measurement of the fetus in the first trimester, up to and including 13 weeks and 6 days of gestation; gestational age assessment based on measurement of the crown‐rump length has an accuracy of 5–7 days (Committee on Obstetric Practice American Institute of Ultrasound in Medicine Society for Maternal–Fetal Medicine, 2017). Second‐trimester ultrasound scans conducted between 18 and 28 weeks, despite allowing a detailed foetal anatomical evaluation, introduce greater variability and complexity and are less accurate for gestational age assessment (Committee on Obstetric Practice American Institute of Ultrasound in Medicine Society for Maternal–Fetal Medicine, 2017); in the first part of the second trimester (up to 22 weeks) ultrasound has an accuracy of 7–10 days, and between 22 and 28 weeks it has an accuracy of approximately 10–14 days (Committee on Obstetric Practice American Institute of Ultrasound in Medicine Society for Maternal–Fetal Medicine, 2017). For the seven trials that used ultrasound for gestational age assessment, the mean gestational age at enrollment (and the time of the ultrasound scan) varied between 9 and 17 weeks, which is in line with WHO's recommendation to perform one ultrasound scan before 24 weeks of gestation to estimate gestational age, improve detection of foetal anomalies and multiple pregnancies, reduce induction of labour for pregnancy thought to be postterm and improve a woman's pregnancy experience (WHO, 2022).

Although recognized as the best method of gestational age assessment, ultrasound examinations may be impractical in large trials conducted in rural areas, or areas where there is a lack of or late access to antenatal care (ANC). It is noteworthy that the latest estimated coverage of early (first trimester) ANC visits in low‐income countries was only 24% (Moller et al., 2017), and other methods for gestational age dating may be necessary. This is why researchers conducting micronutrient supplementation trials in LMIC developed a pregnancy surveillance system to identify pregnancies early in gestation, involving visits every 5 weeks to the homes of women of reproductive age to ask them about the date of their LMP (and the provision of a calendar where women were instructed to mark the date of the beginning of their menses) and conduct urine‐based pregnancy tests (Christian et al., 2003). This method was validated against crown‐rump length measured in early pregnancy by ultrasound, showing that gestational age varied by a mean of only 2.8 days (Gernand, Paul, et al., 2016); as such, it can be considered a valid measure for estimating gestational age and preterm birth rates (Gernand, Paul, et al., 2016).

Still, the most widely available method for gestational dating in LMIC is the self‐reported or recall (i.e., retrospective collection) of the 1st day of the LMP. This method is based on the assumption that the average menstrual cycle is 28 days in length with ovulation occurring on Day 14, and pregnancy is assumed to last 280 days from the 1st day of the LMP. It has varying levels of accuracy among different populations, and several limitations such as uncertainty of the date due to recall bias, variations in the timing of ovulation and bleeding that can occur for other reasons other than menses (Lynch & Zhang, 2007).

These limitations of the retrospective collection (recall) of the 1st day of the LMP can lead to more errors in the estimation of gestational age, which can affect the estimated prevalence of preterm birth or SGA. Assessment of birthweight is not dependent on gestational age measurements, so there may be less error in estimates of this outcome than is the case for preterm births or SGA. However, it should be noted that errors in any method used to assess gestational age (or weight) were expected to be the same in both MMS and IFA groups of a masked randomized clinical trial. The potential nondifferential measurement error for outcomes (i.e., if errors are the same in both trial arms) would bias the results towards the null (Rothman et al., 2008). Our subgroup analyses are consistent with this established epidemiological principle. In addition, it should be noted that it is not expected that LBW is entirely explained by SGA or through preterm births; both pathways are plausible and may overlap. Not all preterm births lead to LBW infants and some SGA babies may not be LBW.

These results provide new evidence to support the benefit of MMS versus IFA that can be used to inform a reevaluation of the WHO recommendations on antenatal micronutrient supplements (WHO, 2020). We conclude there is no need for additional controlled clinical trials in which early pregnancy ultrasound is used to determine effects on preterm births or SGA because this has already been demonstrated with existing trials. New clinical trials would require substantial resources and time. Further, when considering that there are already 16 existing trials with over 100,000 pregnant women, the inclusion of newly generated results would likely have little impact on the overall effect estimates on birth outcomes.

### 4.2 Assessment of benefits of antenatal micronutrient supplementation by WHO

When reviewing the evidence behind the recommendations for using antenatal MMS between 2016 (WHO, 2016) and 2020 (WHO, 2020), WHO noted that ‘The resulting evidence on effectiveness was found to be largely similar to that evaluated during the 2016 guideline development process, showing an average 12% (9%–14%) reduction in low birth weight with MMS but little difference in effects on low birth weight's component parts (preterm birth or being small for gestational age). When analyses were limited to the 10 trials comparing UNIMMAP MMS with IFA supplements, low birth weight was reduced by 13% (95% CI, 6% to 19%) and small for gestational age was reduced by 9% (2% to 15%) on average’ (Tuncalp et al., 2020). This led to the conclusion that while ‘there may be a limited benefit and little harm in replacing IFA with MMS, the evidence on low birthweight and its component parts (preterm birth and SGA) is difficult to interpret’ (WHO, 2020). While the relatively wide confidence intervals of the effect estimates may have contributed to a conditional recommendation, we would argue that the evidence is consistent and compelling.

First, we believe that a 12% reduction in LBW (or 13% for the UNIMMAP supplements) could lead to meaningful benefits. The latest global estimates indicate that 20.5 million livebirths have a birthweight of less than 2500 g, and 91% of these are born in LMICs, mainly southern Asia (48%) and sub‐Saharan Africa (24%) (Blencowe et al., 2019). Thus, a 12% reduction in LBW observed with MMS (or an even greater reduction in the group of anaemic women [Smith et al., 2017]) has the potential to benefit an estimated 2.2 million infants in LMIC annually. Moreover, an additional gain in weight or duration of pregnancy would likely be beneficial to many more infants even if it does not change their categorization as LBW, preterm or SGA. This is a significant and important benefit, in addition to the benefit conferred by IFA (the comparison group), as LBW is shown to be associated with increased mortality and morbidity (Gu et al., 2017; UNICEF, 2022). The benefits of MMS for preterm births and SGA (observed when gestational age is assessed with the best method(s)) are equally important, as these two outcomes are known to increase mortality risk in and beyond the neonatal period (Katz et al., 2013).

Second, we note that several current WHO recommendations on ANC are based largely on reducing LBW including nutrition education on increasing daily energy and protein intake in undernourished populations, and restricting caffeine intake (WHO, 2016). Both of those interventions had little to no effect, and low or very low‐quality evidence, on preterm birth and SGA. Daily oral IFA supplementation is also recommended to reduce LBW. Per the WHO guidelines the risk reduction of IFA versus placebo on LBW *is not significant* (RR, 0.84; 95% CI, 0.69–1.03) and was graded as low‐certainty evidence, but was nonetheless interpreted as ‘daily iron *may* reduce the risk of low‐birth‐weight neonates’ (WHO, 2016). None of the recommendations that were based on reduction of LBW were conditional on additional research or requested evidence from studies with ultrasound‐based gestational age assessment. In contrast, the risk reduction of MMS versus IFA on LBW *is significant* (overall analysis: RR, 0.88; 95% CI, 0.86–0.91) and was graded as high‐certainty evidence (WHO, 2020).

## 5 CONCLUSION

The present analyses, based on data from the trials used to inform the 2020 WHO guidelines, demonstrated consistent results regarding the effect of MMS versus IFA on birth outcomes regardless of the method of gestational age assessment. The analyses also showed that 11 of those 16 controlled trials used valid methods to assess gestational age, and that the benefits of MMS (in comparison to IFA) in reducing the risk of adverse birth outcomes were more pronounced in those 11 trials—a finding made more relevant by new estimates for the burden of LBW globally. These results provide new evidence to support the validity of findings from existing trials, challenging the need for new efficacy trials using ultrasound for gestational age assessment and providing further support for a transition from IFA to MMS programmes in LMICs. Future convenings of the WHO guideline development group should consider these findings, in addition to other recent analyses demonstrating comparable effects of MMS (vs. IFA) on maternal anaemia outcomes (Gomes et al., 2022), when updating recommendations related to using MMS during pregnancy.

## AUTHOR CONTRIBUTIONS

Filomena Gomes, Robert E. Black, Parul Christian and Emily R. Smith conceptualized and designed the analyses. Filomena Gomes and Ziaul Rana extracted the data. Filomena Gomes carried out the analyses and drafted the manuscript. All authors contributed to the interpretation of the analyses, critically revised the manuscript, and approved its final version.

## CONFLICT OF INTEREST STATEMENT

The authors declare no conflict of interest.

## Supporting information

Supporting information.

## Data Availability

The data that support the findings of this study are available from the corresponding author upon reasonable request.
