# Supplementary material for: Antenatal multiple micronutrient supplements versus iron‐folic acid supplements and birth outcomes: Analysis by gestational age assessment method
Source: Matern Child Nutr. 2023 Mar 31;19(3):e13509. doi: 10.1111/mcn.13509 (PMC10262881; doi:10.1111/mcn.13509)
Supplement: Supplementary file 1 — Supporting information. [file MCN-19-e13509-s001.docx]

**APPENDICES**

Appendix Table 1 – Method used for gestational age assessment in all the 16 trials included for analysis in the 2020 WHO guidelines

| **Study (author, year)** | **Group (1-3) according to gestational age assessment method** | **Method used to assess gestational age** | **Evidence of method used to assess gestational age** |
| --- | --- | --- | --- |
| Ashorn, 2015 | 1 | Ultrasound | “The target population comprised pregnant women who came for antenatal care at any of the study clinics during the enrollment period and met the following inclusion criteria: ultrasound confirmed pregnancy of no more than 20 completed gestation weeks,”…" and "We calculated the duration of pregnancy by adding the time interval between enrollment and miscarriage or delivery to the ultrasound-determined gestational age at enrollment" (Ashorn et al., 2015) |
| Bhutta, 2009 | 1 | Ultrasound | For all women who were suspected to be pregnant (...) they were invited to participate in the screening examination and ultrasound confirmation of pregnancy. (...) A trained obstetric ultrasonographer performed an examination of all eligible women who agreed to the examination within 7 days of identification of a suspected pregnancy to confirm and date the pregnancy. Those with a confirmed pregnancy at less than 16 weeks of gestation were invited to participate in the trial. (.”.)" and "Gestational age was determined by maternal last menstrual period and ultrasound scan dating" (Bhutta et al., 2009) |
| Christian, 2003 | 2 | Date of last menstruation + pregnancy surveillance with urine test | "All women of reproductive age were first screened by 426 local female workers (“sector distributors”) to assess how likely it was that they would become pregnant over the next 12 months. Women who were currently pregnant, breast feeding a baby < 9 months old, menopause, sterilized, or widowed were excluded. Remaining women were registered and visited by sector distributors at home every five weeks for a year and were asked if they had menstruated in the previous month. If not, women underwent a urine test (human chorionic gonadotrophin antigen; Clue, Orchid Biomedical Systems, Goa, India) to ascertain pregnancies. Throughout the trial, women were added to the registry and pregnancy surveillance when they married." and "Gestational age was calculated from the reported first day of the last menstrual period, obtained at the baseline interview, and checked against the week of the positive pregnancy test and prospectively collected histories of menstruation" (Christian et al., 2003) |
| Dewey 2009 (Adu-Afarwuah 2015) | 1 | Ultrasound | "Potential participants were pregnant women attending antenatal clinics" (…) and “women whose gestational age at screening was based on fundal height and was <18 wk underwent an ultrasound scan immediately after enrollment”(Adu-Afarwuah et al., 2015).  A direct discussion with the study authors confirmed that ultrasound was used for determining duration of gestation. |
| Friis, 2004 | 3 | Date of last menstruation | "Gestational age at recruitment and gestational length of pregnancy were calculated from the first day of the last menstruation or estimated by using fundus height" (Friis et al., 2004) |
| Kaestel, 2005 | 3 | Date of last menstruation | "Gestational age at inclusion was computed as the number of weeks from the first day of the last menstruation until date of enrolment" (Kaestel et al., 2005) |
| Liu, 2013 | 3 | Date of last menstruation | "To be eligible, pregnant women recorded dates of their menstruation for 2 or more months before they became pregnant, (...) and "In addition, having monitored their menstrual cycle for more than 2 mo before enrollment to calculate the exact gestation age, women were cognizant of their last menstrual period and prenatal health" (Liu et al., 2013).  A direct discussion with the study authors confirmed that gestational age was based on the first day of the last menstrual period. |
| Moore, 2009 (Johnson 2017) | 1 | Ultrasound | “At the three prenatal clinic visits, fetal biometry was assessed via ultrasound using a Siemens ACUSON Antares Ultrasound Imaging System (Siemens Medical Solutions United States of America (USA) Inc; California, USA with a CH6–2 (5.71 MHz) transducer). Using the built‐in equations, the purpose of the fetal biometry measurements taken at booking was to estimate gestational age. This estimation was based on crown‐rump length (CRL) if gestational age was <12 weeks, or bi‐parietal diameter (BPD) if CRL was too large to be accurately measured or gestational age was ≥12 weeks. If CRL was not yet measurable, gestational age was estimated according to the size of the gestational sac, and the woman’s booking visit was rescheduled for 12 weeks gestation” (Johnson et al., 2017). |
| Osrin, 2005 | 1 | Ultrasound | “We estimated gestational age on the basis of transabdominal ultrasound fetal biometry with an Aloka SSD 900 with obstetric probe (Tokyo, Japan). In pregnancies less than 13 weeks and 6 days, we used crown-rump length and the chart of Robinson. Between 14 and 20 weeks, we used biparietal diameter and head circumference and the charts of Chitty” (Osrin et al., 2005) |
| Roberfroid, 2008 | 1 | Ultrasound | "A consultant obstetrician performed trans-abdominal ultrasound fetal biometry as soon as possible after inclusion of a subject in the study to assess gestational age" (Roberfroid et al., 2008) |
| SUMMIT, 2008 (Shankar, 2008) | 3 | Date of last menstruation | "Gestational age: the duration of pregnancy in weeks calculated from the first day of last menstrual period" (Supplementation et al., 2008) |
| Sunawang, 2009 | 3 | Date of last menstruation | "The mean gestational age at baseline, calculated from recall of the last menstruation date" (Sunawang et al., 2009) |
| Tofail 2008 | 1 | Ultrasound “ | "With the use of the Health and Demographic Surveillance System, all pregnant women were identified within 6–8 wk of conception through regular home visits, and their pregnancies were confirmed by urine tests and ultrasonography" (Tofail et al., 2008)  “A woman who tested positive was encouraged to visit the ICDDR,B clinic as soon as possible, preferably at 8 to 10 weeks of pregnancy, where an ultrasound examination was offered. The following eligibility criteria had to be met for enrollment: viable fetus, gestational age of less than 14 weeks by ultrasound examination, etc.”(Persson et al., 2012) |
| West, 2014 | 2 | Date of last menstruation + pregnancy surveillance with urine test | “Pregnancy surveillance began on...”, “Using a preexisting list of resident women, 596 female sector staff visited all households to confirm eligibility and enlist newly married women aged 12 to 45 years living with their husbands. Each year women were given a wall calendar on which to mark the first day of each menstrual period. Thereafter, every 5 weeks, staff revisited households to continue enlisting newlyweds and ask women about menstruation in the past 30 days. Women reporting amenorrhea were asked the date of the first day of their last menstrual period, checked against marked calendars, and offered a urinary human chorionic gonadotropin test (Orchid) to confirm pregnancy. Women with positive test results were visited within days by team leaders who provided information about the trial and requested participation." and "Gestational age at pregnancy recruitment and outcome were calculated in the data center, based on the first day of last menstrual period immediately before positive urine test results" (West et al., 2014) |
| Zagre, 2007 | 2 | Date of last menstruation + pregnancy surveillance with urine test | "Women were eligible to participate in the study if they lived in one of the selected villages and if they had experienced amenorrhea for less than 12 weeks. Within their areas, traditional midwives were in charge of identifying women of reproductive age and those among them who had missed their menstruation at least once. After the nurse in charge of the integrated health center confirmed the pregnancy with a pregnancy test, the woman was informed of the study objectives,…” (Zagre et al., 2007)  A direct discussion with the study authors confirmed that there was an active pregnancy surveillance system with pregnancy tests to confirm pregnancies, and the gestational age assessment was based on the first day of the last menstrual period |
| Zeng, 2008 | 2 | Date of last menstruation + pregnancy surveillance with urine test | "Trained village doctors obtained informed verbal consent for pregnancy monitoring, and consenting women were visited every month and asked about the date of their last menstrual period. Women with periods delayed by more than five days had a urine pregnancy test, and confirmed pregnancies were reported to the township maternal and child health worker" and "Gestational age at birth was measured as completed days based on the first day of the last menstrual period" (Zeng et al., 2008) |

Appendix Table 2 – Sensitivity analysis limited to the trials that used UNIMMAP supplements in the intervention arm (excluding the West 2014 trial): subgroup analyses for preterm, small for gestational age, and low birthweight, according to gestational age assessment method

| **Method used for gestational age assessment** | **Effect of MMS versus IFA on low birthweight** | **Effect of MMS versus IFA on preterm** | **Effect of MMS versus IFA on small for gestational age** |
| --- | --- | --- | --- |
|  | **RR (95%CI)** | **RR (95%CI)** | **RR (95%CI)** |
|  | *n trials* | *n trials* | *n trials* |
| **Overall analyses** | **0.87 (0.81-0.94)** | 1.00 (0.96-1.03) | **0.91 (0.85-0.98)** |
|  | *10 trials* | *10 trials* | *9 trials* |
| **Subgroup analyses (3 groups)** |  |  |  |
| 1 - ultrasound | **0.87 (0.79-0.96)** | 0.93 (0.79-1.11) | 0.91 (0.81-1.01) |
|  | *4 trials* | *4 trials* | *4 trials* |
| 2 - prospective date of LMP collection and confirmation of pregnancy by urine test | 0.87 (0.69-1.10) | 1.03 (0.92-1.15) | 0.87 (0.75-1.02) |
|  | *2 trials* | 2 | *2 trials* |
| 3 - recall of date of last menstrual period | **0.87 (0.77-0.99)** | 1.00 (0.96-1.04) | 0.94 (0.84-1.05) |
|  | *4 trials* | *4 trials* | *3 trials* |
| **Subgroup analyses (2 groups)** |  |  |  |
| 1 – best methods (ultrasound and prospective LMP collection with pregnancy test) | **0.87 (0.79-0.95)** | 0.99 (0.90-1.09) | **0.89 (0.82-0.98)** |
|  | *6 trials* | *6 trials* | *6 trials* |
| 2 – recall of date of last menstrual period | **0.87 (0.77-0.99)** | 1.00 (0.96-1.04) | 0.94 (0.84-1.05) |
|  | *4 trials* | *4 trials* | *3 trials* |

*LMP = last menstrual period*

*Statistically significant results are identified in* ***bold***

All *p* values of Chi^2^ tests for heterogeneity across subgroups were > 0.05*.*

Appendix Table–3 - Sensitivity analysis limited to the trials that used UNIMMAP supplements in the intervention arm (including the West 2014 trial): subgroup analyses for preterm, small for gestational age, and low birthweight, according to gestational age assessment method

| **Method used for gestational age assessment** | **Effect of MMS versus IFA on low birthweight** | **Effect of MMS versus IFA on preterm** | **Effect of MMS versus IFA on small for gestational age** |
| --- | --- | --- | --- |
|  | **RR (95%CI)** | **RR (95%CI)** | **RR (95%CI)** |
|  | *n trials* | *n trials* | *n trials* |
| **Overall analyses** | **0.88 (0.85-0.91)** | 0.96 (0.89-1.04) | **0.97 (0.96-0.99)** |
|  | *11 trials* | *11 trials* | *10 trials* |
| **Subgroup analyses (3 groups)** |  |  |  |
| 1 - ultrasound | **0.87 (0.78-0.96)** | 0.93 (0.79-1.11) | 0.91 (0.81-1.01) |
|  | *4 trials* | *4 trials* | *4 trials* |
| 2 - prospective date of LMP collection and confirmation of pregnancy by urine test | **0.88 (0.85-0.91)** | 0.95 (0.81-1.11) | 0.96 (0.90-1.03) |
|  | *3 trials* | *3 trials* | *3 trials* |
| 3 - recall of date of last menstrual period | **0.87 (0.77-0.99)** | 1 (0.96-1.04) | 0.94 (0.84-1.05) |
|  | *4 trials* | *4 trials* | *3 trials* |
| **Subgroup analyses (2 groups)** |  |  |  |
| 1 – best methods (ultrasound and prospective LMP collection with pregnancy test) | **0.88 (0.85-0.91)** | 0.94 (0.84-1.05) | 0.98 (0.96-1.00) |
|  | *7 trials* | *7 trials* | *7 trials* |
| 2 - recall of date of last menstrual period | **0.87 (0.77-0.99)** | 1.00 (0.96-1.04) | 0.94 (0.84-1.05) |
|  | *4 trials* | *4 trials* | *3 trials* |

*LMP = last menstrual period*

*Statistically significant results are identified in* ***bold***

All *p* values of Chi^2^ tests for heterogeneity across subgroups were > 0.05*.*

Appendix Figure 1 – Effect of MMS vs IFA on low birthweight: subgroup analysis by method of gestational age assessment (2 groups)


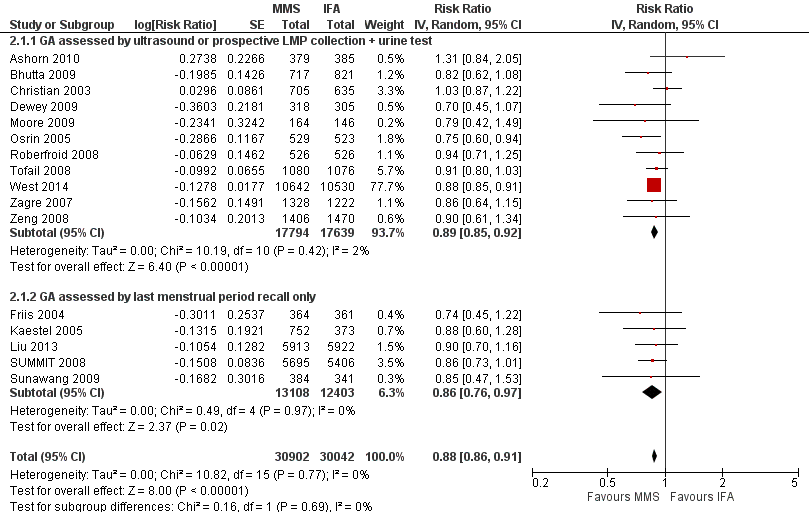


*MMS = multiple micronutrient supplementation; IFA = iron and folic acid supplementation; GA = gestational assessment; LMP = (first day of) last menstrual period*

Appendix figure 2 – Effect of MMS vs IFA on preterm birth: subgroup analysis by method of gestational age assessment (2 groups)
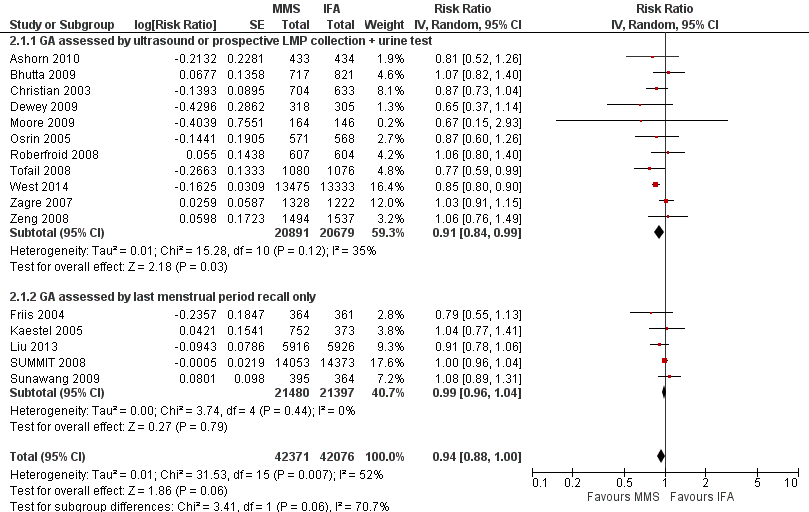


*MMS = multiple micronutrient supplementation; IFA = iron and folic acid supplementation; GA = gestational assessment; LMP = (first day of) last menstrual period*

Appendix figure 3 – Effect of MMS vs IFA on small for gestational age: subgroup analysis by method of gestational age assessment (2 groups)


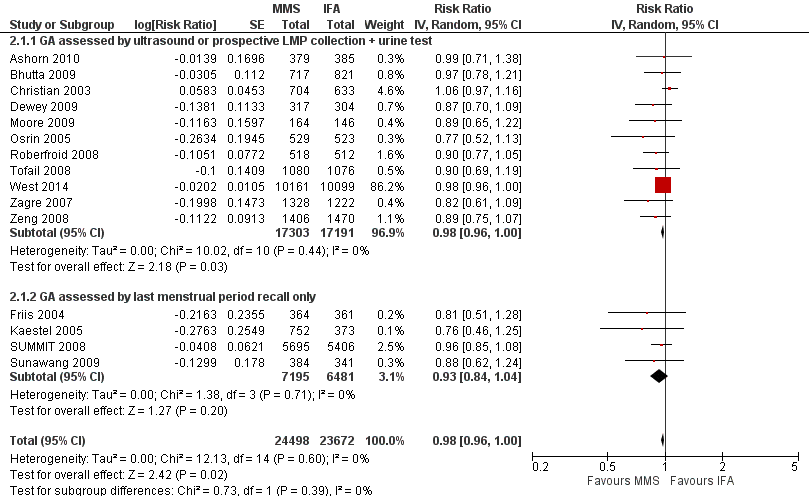


*MMS = multiple micronutrient supplementation; IFA = iron and folic acid supplementation; GA = gestational assessment; LMP = (first day of) last menstrual period*
